# Diagnostic accuracy of metagenomic next-generation sequencing in diagnosing infectious diseases: a meta-analysis

**DOI:** 10.1038/s41598-022-25314-y

**Published:** 2022-12-05

**Authors:** Jian Liu, Qiao Zhang, Yong-Quan Dong, Jie Yin, Yun-Qing Qiu

**Affiliations:** 1 Department of Intensive Care Unit, the First Affiliated Hospital, College of Medicine, Zhejiang University, Hangzhou, Zhejiang China; 2 Department of Clinical Pharmacy, Zhejiang Provincial Key Laboratory for Drug Evaluation and Clinical Research, the First Affiliated Hospital, College of Medicine, Zhejiang University, Hangzhou, Zhejiang China; 3 Department of Respiratory Disease, Yinzhou No.2 Hospital, Ningbo, Zhejiang China; 4 Department of Colorectal Medicine, The Cancer Hospital of the University of Chinese Academy of Sciences (Zhejiang Cancer Hospital), Institute of Basic Medicine and Cancer (IBMC), Chinese Academy of Sciences, Hangzhou, Zhejiang China; 5 Department of Infectious Diseases, The First Affiliated Hospital, College of Medicine, Zhejiang University, Hangzhou, Zhejiang China

**Keywords:** Microbiology, Diseases, Health care

## Abstract

Many common pathogens are difficult or impossible to detect using conventional microbiological tests. However, the rapid and untargeted nature of metagenomic next-generation sequencing (mNGS) appears to be a promising alternative. To perform a systematic review and meta-analysis of evidence regarding the diagnostic accuracy of mNGS in patients with infectious diseases. An electronic literature search of Embase, PubMed and Scopus databases was performed. Quality was assessed using the Quality Assessment of Diagnostic Accuracy Studies-2 tool. Summary receiver operating characteristics (sROC) and the area under the curve (AUC) were calculated; A random-effects model was used in cases of heterogeneity. A total of 20 papers were eligible for inclusion and synthesis. The sensitivity and specificity of diagnostic mNGS were 75% and 68%, respectively. The AUC from the SROC was 85%, corresponding to excellent performance. mNGS demonstrated satisfactory diagnostic performance for infections and yielded an overall detection rate superior to conventional methods.

## Introduction

Infectious diseases are a leading cause of morbidity and death worldwide. However, early detection of pathogens may be challenging in many clinical scenarios. Moreover, many common pathogens are difficult or impossible to detect using conventional microbiological tests (e.g. culture, smears, immunological tests and polymerase chain reaction (PCR) assays), which makes precise diagnosis challenging. Culture methods are time-consuming and have strict limitations. Smears, immunological tests and multiplex PCR assays will only test for a specific pathogen that must be identified by the clinicians before the test is performed^1^. The administration of broad-spectrum antibiotics in the absence of pathogen identification, despite comprehensive testing methods, frequently confounds specific diagnoses, which could lead to more toxic and less effective antimicrobial therapy^2^.

Metagenomic next-generation sequencing (mNGS) is a high-throughput method that can directly detect pathogens (i.e., bacterial species) in clinical specimens and analyze functional genes without the need to pre-select target sequences^3^. It is especially suitable for novel, rare, and atypical etiologies of complicated infectious diseases. Due to characteristics of speed, sensitivity, culture-independent, hypothesis-free, and unbiased pathogen detection, mNGS may become a routine diagnostic tool, partly replacing more traditional detection methods^4^. Some investigators have even decided to upgrade their model, known as ‘Microbial Index of Pathogenic Bacteria’, by implementing whole metagenome sequencing data for species and strain- level identification of patho-genic bacteria^5^. To date, mNGS has been applied in the diagnosis of pathogens in bloodstream infections^6,7^, respiratory tract infections^8,9^, tuberculosis^10^, meningitis and encephalitis^11,12^. However, these studies were limited by small sample sizes. As such, we aimed to perform a systematic accuracy review of diagnostic tests and a meta-analysis to identify, quality appraise, and synthesize the available evidence to inform the implementation of mNGS in diagnosing infectious diseases.

## Methods

### Literature search

Results of the present systematic review and meta-analysis are reported in accordance with the Preferred Reporting Items for a Systematic Review and Meta-analysis of Diagnostic Test Accuracy studies (PRISMA-DTA)^13^. A comprehensive electronic literature search of Embase, PubMed and Scopus databases was performed for relevant studies published up to December 31, 2021. The medical subject heading (i.e., ‘MeSH’) search terms included 'infection' and 'Metagenomic Next-Generation Sequencing'. The reference lists of retrieved studies were also manually searched for additional, possibly eligible studies. Three reviewers independently screened the titles, and abstracts and obtained the full-text of potentially relevant studies; any disagreements were resolved by consensus discussion.

### Inclusion and exclusion criteria

Cross-sectional and cohort studies including patients with clinically suspicious infection (including meningitis, bacteremia, fungemia, osteomyelitis, septic arthritis) for whom diagnostic test accuracy data for mNGS were included. Only English language articles were eligible. No restrictions were imposed on the age of the study population.

Studies reporting insufficient data to construct a 2 × 2 table (true positive, false positive, true negative, and false negative), those based on non-human samples, investigations reporting duplicate information already reported in other publications; those not reporting the reference infection diagnostic criteria; or reporting one specific pathogene and abstracts, conference presentations, case reports and letters were excluded.

### Data extraction

Data were independently extracted by two reviewers using a standardized protocol and prespecified data extraction forms for diagnostic test accuracy studies^14^. Disagreements were resolved by a third investigator. Information regarding study characteristics (including population, period, design, country, and sample size) was extracted.

### Quality assessment

The quality of the included studies was independently assessed by two reviewers, using the revised Quality Assessment of Diagnostic Accuracy Studies-2 tool^15^.

### Statistical analysis

For each study, pooled specificity, pooled sensitivity, pooled negative predictive value (NPV), and pooled positive predictive value (PPV) were calculated based on a bivariate meta-analysis model^16^. They are presented as graphical representations in which the boxes mark the values and the horizontal lines represent the confidence intervals (CIs). A summary receiver operating characteristic curve (sROC) was drawn, and the area under the curve (AUC) was calculated to determine the performance of a diagnostic test^17^. The criteria for AUC classification were as follows: 0.50 (failure), 0.60–0.70 (poor), 0.70–0.80 (fair), 0.80–0.90 (good) and 0.90–1 (excellent). The Q* index and corresponding standard error (SE), is an additional measure which is the point on the sROC curve closest to the ideal left top-left corner (where summary sensitivities (SN) and summary specificities (SP) meet).

Heterogeneity was evaluated by calculating the I^2^^18^ statistic. DerSimonian and Laird random effects models^19^, which include both between and within study heterogeneity, were used to generate summary SP, SN, negative likelihood ratios (− LR), positive likelihood ratios (+ LR) and diagnostic odds ratio (DOR). Heterogeneity was also assessed using forest plots of sensitivity and specificity across studies for variability of study estimates in the hierarchical sROC model (meta-regression). A Cochrane’s-Q *p* < 0.10 and I^2^ > 50% indicated significant heterogeneity, of SN and SP and LRs, respectively. Furthermore, the risk of bias in the included studies was assessed by using the Quality Assessment of Diagnostic Accuracy Studies-2 (QUADAS–2) tool^15^. Publication bias was assessed by using a funnel plot and Deeks test^20^. Statistical analysis was performed using MetaDisc version 1.4, Stata version 12.0 (StataCorp LLC, College Station, TX, USA), and Review Manager 5 (version 5.3) (R Foundation for Statistical Computing, Vienna, Austria)^21^.

## Results

### Characteristics of the included studies

**Figure 1:**
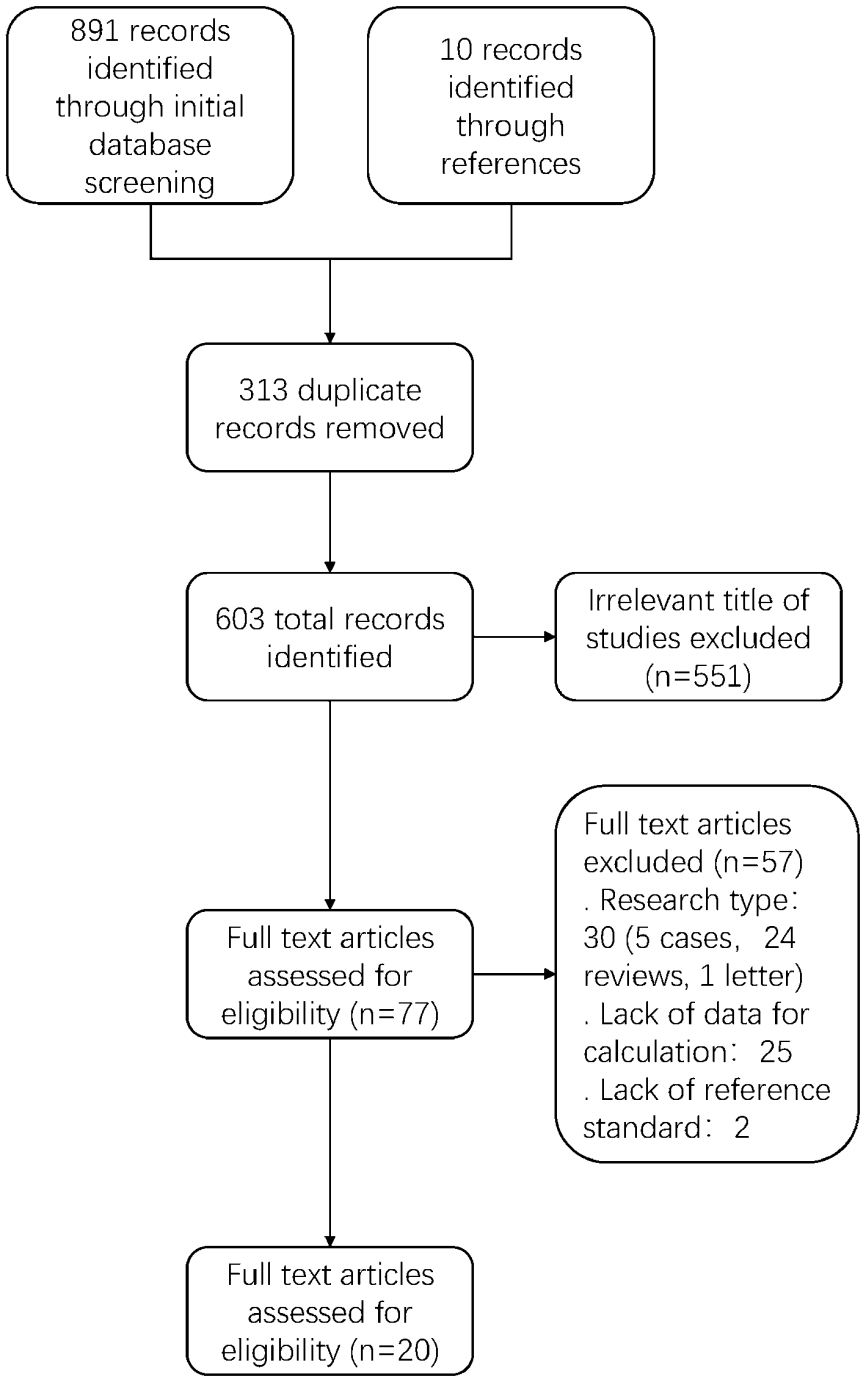
Flow diagram of study selection process.

After removing duplicate publications and references checking for additional, potentially eligible studies, a total of 891 studies were screened. Of these, 77 separate publications underwent full text review, resulting in 20 studies included in this systematic review. The study selection process is illustrated in the PRISMA flow diagram (Fig. 1). Six studies were performed in high-income countries, whereas 14 were conducted in low and middle-income countries. The study included 2716 participants. Twelve of the studies were retrospective, and eight were prospective in design. Among the enrolled studies, participants were predominantly adults. The included studies were published between 2017 and 2021. (Table 1).

**Table 1:** Characteristics of Included Studies.

	Research type	Reference standard	Participants	Age	Country	Sample type
Zhang^32^	Retrospective	Conventional test	135	Paediatric	China	Blood/CSF
Wang^30^	Retrospective	Conventional test	55	Adults	China	Pulmonary biopsy/BALF
Rossoff^33^	Retrospective	Conventional test	79	Pediatric	USA	Plasma
Miller^34^	Retrospective	Conventional test	95	Pediatric + adult	USA	CSF
Blauwkamp^6^	Prospective	Conventional test	348	Adults	USA	Plasma
Miao^24^	Retrospective	Conventional test	511	Adults	Chian	Clinical specimens^a^
Madi^35^	Retrospective	Conventional test	86	Adults	Kuwait	Respiratory samples^b^
Parize^36^	Prospective	Conventional test	101	Adults	France	Plasma/nasopharyngeal swabs/ biological fluid
Xing^28^	Prospective	Conventional test	213	Adults	Chian	CSF
Wang^37^	Prospective	Clinical diagnosis	63	Adults	Chian	Joint fluid
Chen^38^	Retrospective	Conventional test	235	Adults	Chian	BALF
Lian^39^	Retrospective	Clinical diagnosis	51	Adults	Chian	BALF
Peng^40^	Retrospective	Clinical diagnosis	49	Adults	Chian	BALF
Sun^41^	Prospective	Conventional test	44	Adults	Chian	BALF
Zhou^42^	Prospective	Conventional test	159	Adults	Chian	BALF
Chen^43^	Prospective	Conventional test	162	Adults	Chian	BALF
Jing^44^	Retrospective	Clinical diagnosis	209	pediatric + adult	Chian	Plasma
Ogawa^45^	Retrospective	Conventional test	23	Adults	Japan	Tissue
Lee^46^	Retrospective	Clinical diagnosis	54	Pediatric	USA	Plasma
Cai^47^	Prospective	Clinical diagnosis	44	Adults	China	Periprosthetic tissues

a: Specimens included bronchoalveolar lavage fluid (BALF), cerebrospinal fluid (CSF), sputum, pleural fluid, tissue, pus, blood, ascetic fluid, bile, secretion, urine, herpes fluid, bone marrow, throat swab, pericardial fluid and saliva; b: The respiratory samples included nasopharyngeal aspirates/wash, nasopharyngeal swab, BALF, tracheal aspirates, sputum, throat swabs, and nasal swabs.

### Risk of bias

**Figure 2:**
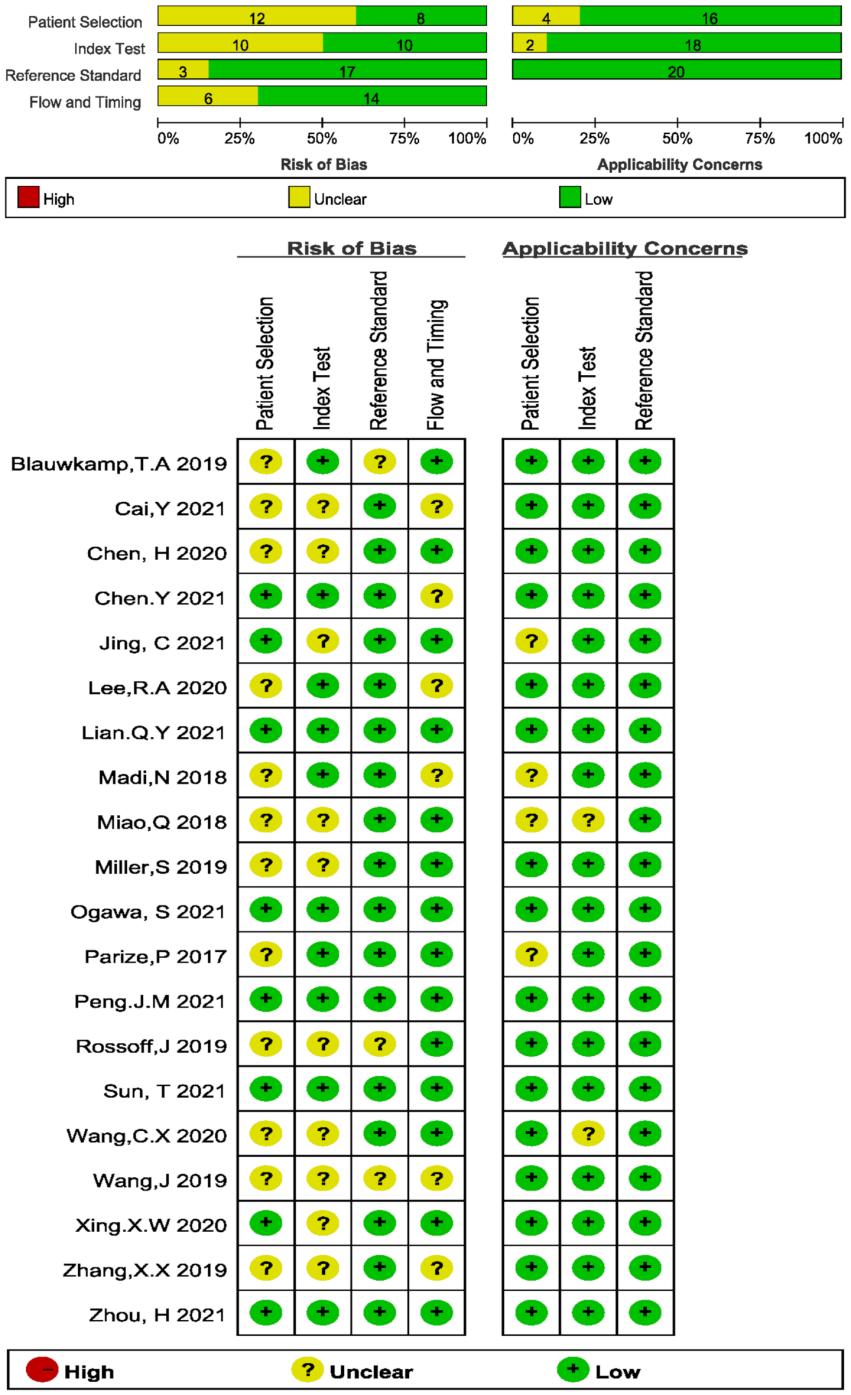
Risk of bias and applicability concerns summary.

The risk of bias and applicability concerns according to the QUADAS-2 tool are shown in Fig. 2. All studies demonstrated unclear or low risks of bias.

### Meta‑analysis

#### Heterogeneity test

No correlation was found between sensitivity logarithm and 1-specificity logarithm (Spearman correlation 0.0345 (*p* = 0.092)). Analysis revealed no threshold effect among the included studies. As is common with meta-analyses investigating results of diagnostic accuracy research, remarkable heterogeneity was present, with sensitivity and specificity estimates varying widely. The Cochran-Q for the pooled DOR was 207.82, (*p* = 0.00, I^2^ = 88.5%). This suggests that a non-threshold effect was the cause of heterogeneity and a random effect model was used in further analysis.

#### Random effect model analysis results

**Figure 3:**
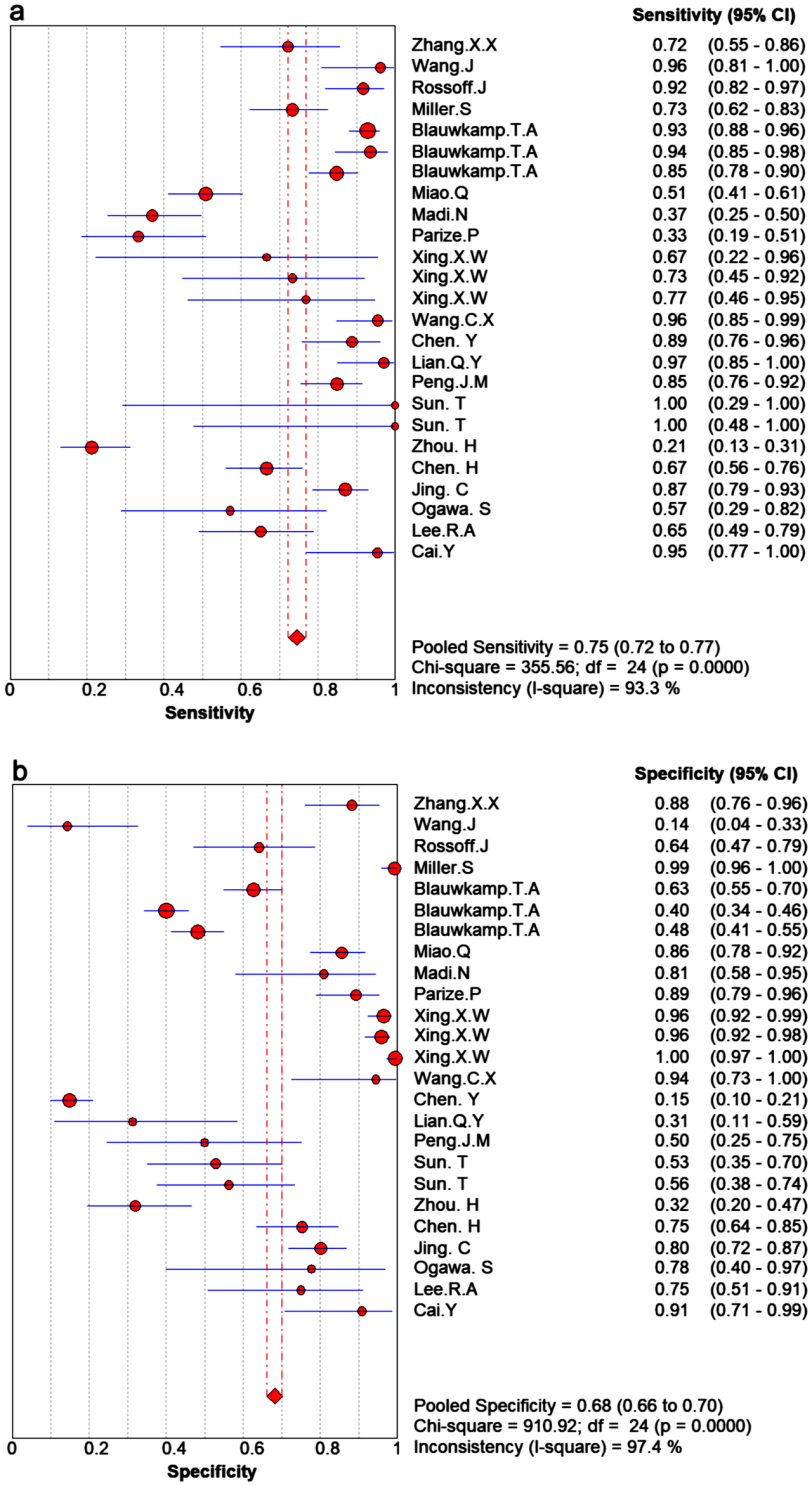
Forest plot of estimates results: (**a**) Sensitivity; (**b**) Specificity.

**Figure 4:**
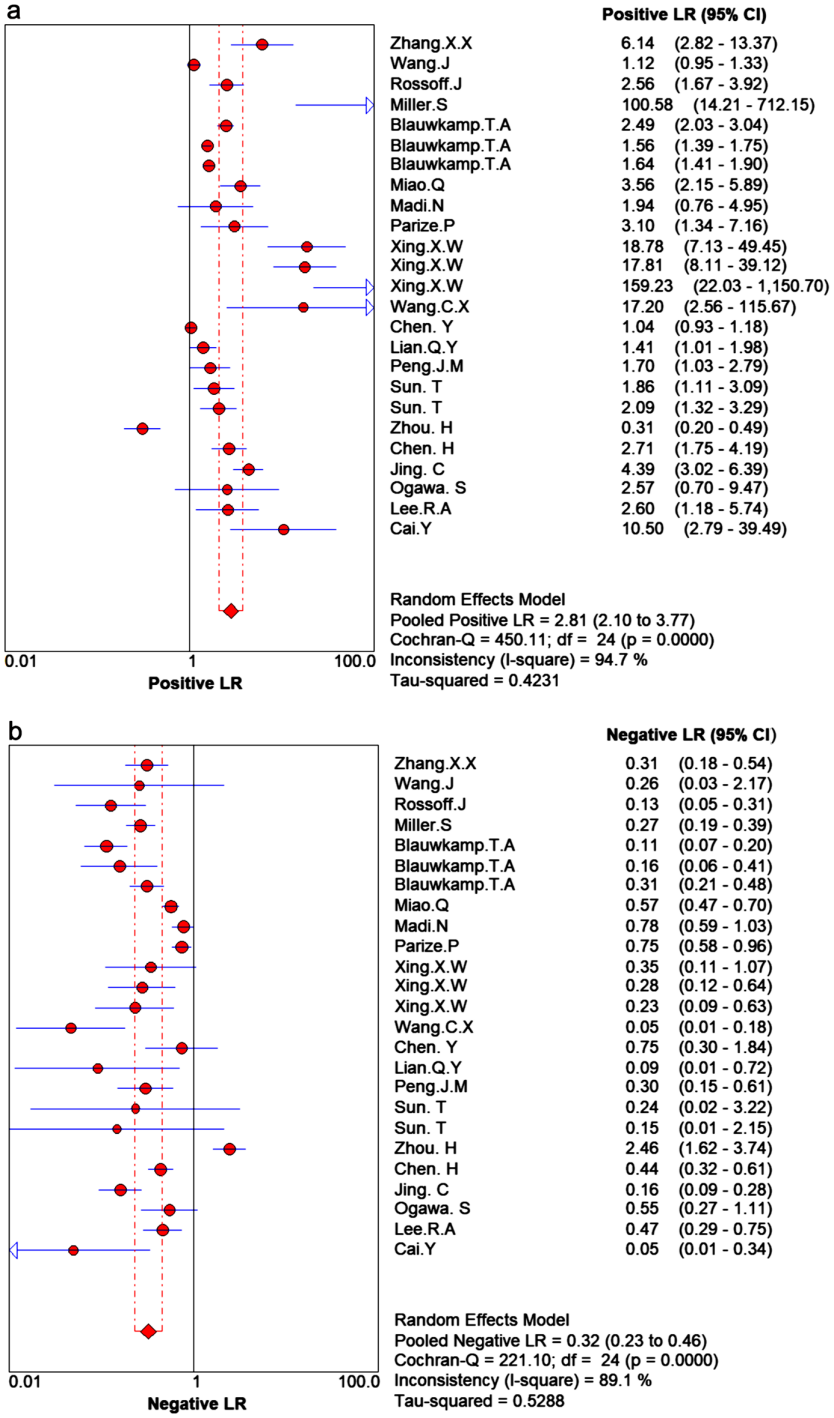
(**a**) Positive likelihood ratio; (**b**) Negative likelihood ratio.

The reported diagnostic sensitivity of the mNGS in infectious diseases ranged between 21% and 100% (Fig. 3a), and the reported specificity ranged from 14% to 100% (Fig. 3b). The pooled summary sensitivity reached 75% (95% CI 72–77%, I^2^ = 93.3%) (Fig. 3a) and pooled summary specificity was computed to 68% (95% CI: 66%–70%, I^2 ^= 97.4%) (Fig. 3b), indicating significant heterogeneity. The pooled positive LR was 2.8 (95% CI: 2.1–3.77) and the pooled negative LR was 0.32 (95% CI: 0.23–0.46) (Fig. 4a,b).

#### Subgroup analysis

Subgroup analysis was also performed to explore the influence of different reference standards in the final result (Supplementary Fig. 1a–d). Two subgroups were formed based on two reference standards: conventional testing and clinical diagnosis. The results confirmed consistent performance.

#### Heterogeneity analysis

Four components, including “gold standard”, “experimental design”, “age” and “country income” were considered in the meta-regression analysis to explore potential risk of bias. Unfortunately, none of these components exhibited heterogeneity. Due to failure to extract more comprehensive data from the research, it was not further analyzed.

#### Evaluation of diagnostic accuracy

**Figure 5:**
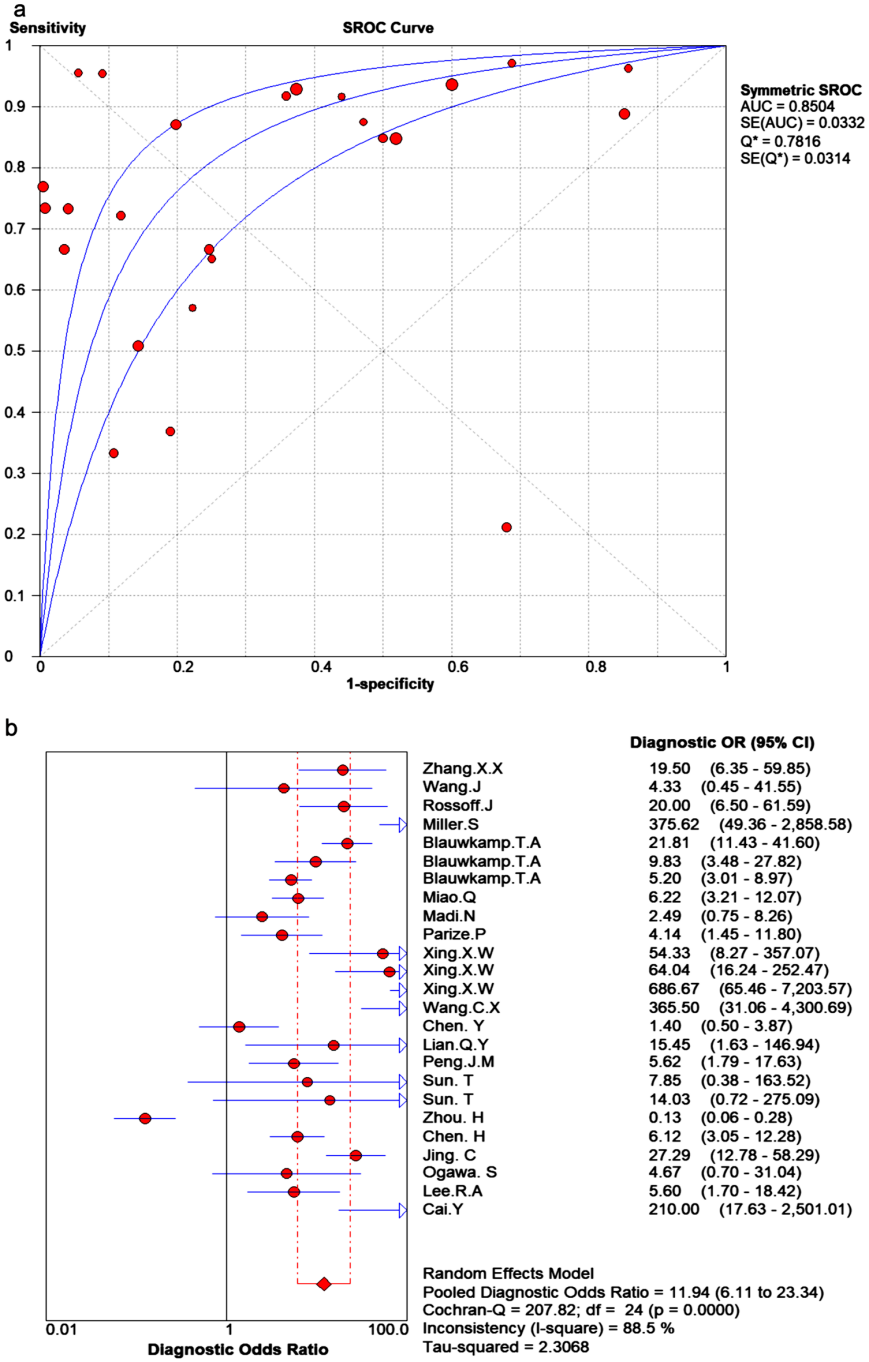
Summary ROC curves.

SROC curves for the mNGS in infectious diseases are presented in Fig. 5a. This figure illustrates the relationship between sensitivities and 1-specificity for the included studies in the pooled analyses. The AUC was considered excellent (AUC = 0.85 (SE = 0.03)). The point at which sensitivity and specificity were equal (Q*) was 0.78 (SE = 0.03). The pooled DOR was 11.94 (95% CI: 6.11–23.34) (Fig. 5b).

### Publication bias

Deek’s test yielded no evidence of publication bias (*P* = 0.795). (Supplementary Fig. 2).

## Discussion

To our knowledge, the present meta-analysis was the first to systematically review the use of mNGS in diagnosing infectious diseases. Conventional techniques for the detection of pathogens are largely target-dependent tests, which detect a limited number of micro-organisms. However, NGS-based metagenome approaches are target independent and can detect unknown pathogens^22^. Using the pooled estimate of 75% (95% CI: 72–77%, I^2 ^= 93.3%)) at median specificity 68% (95% CI: 66–70%, I^2^ = 97.4). The AUC 85%, which reflected infection using mNGS, was classified as excellent performance.

The DOR reflects the relationship between the diagnostic test and the relevant disease. The pooled DOR was 11.94, reflecting diagnostic efficacy of mNGS in infectious diseases. The pooled positive LR was 2.81 (95% CI: 2.1–3.77), which reflects that the risk of developing the disease was 2.81 times that of not having the disease when the results of next generation sequencing being positive. The pooled negative LR was 0.32 (95% CI: 0.23–0.46), which reflects that the risk of developing the disease was 0.32 times that of not having the disease when the results of NGS are negative. The sROC curve reflects merge indicators of the sensitivity and specificity. The AUC for sROC was 0.85, which reflected high diagnostic efficiency.

Some studies^23–26^ demonstrated that mNGS had diagnostic advantages over conventional methods for patients treated with empirical antibiotics before sample collection. The use of empirical antibiotics would significantly lower the detection rate of conventional methods by approximately 20%, while mNGS is not affected^23^. The reason may likely be due to the fact that culture methods require the existence of live pathogens and, therefore, are easily influenced by the administration of antimicrobials. On the other hand, high-throughput sequencing needs only to identify DNA fragments of microorganisms, which may explain its relatively higher detection rate after antimicrobial treatment. Moreover, it can shorten turnaround time and detect pathogens without bias^27^.

NGS also has shortcomings. First, it is not sensitive for intracellular bacteria and fungi in difficulty obtaining circulatory genome DNA^23,28^. RNA viruses require reverse transcription before deep sequencing and the amount of DNA segments may be reduced^23^. Different NGS technique may introduce bias (Supplementary Table 1). Second, mNGS is relatively expensive. Third, the criteria for diagnosing single pathogens are unclear, and are mainly based on the relative abundance of pathogens, the coverage rate or unique reads of pathogens^8,29^. In addition, given the untargeted nature of mNGS, background interference is a fairly common limitation.

Our study also had limitations. The first of which was considerable heterogeneity, the sources of which were extensively explored. Meta-regression results revealed that “experimental design” and “age” may have been the cause of heterogeneity. Another factor that needs to be considered is the clinical heterogeneity exhibited in the included studies such as the number of patients, antibiotic treatment, sampling methods, different reference standards and other unknown factors such as technical variations (e.g. sequencing strategies and platforms), sequence profiling software, prediction models, and batch effects. Second, the number of patients in two studies^30,31^ was relatively small, which may have reduced our statistical power. Third, no fourfold contingency tables were feasible for most of the studies because some of the necessary data were calculated based on reported sensitivity and specificity. Fourth, limiting the search strategy to English language publications could have potentially missed some studies. Finally, the included studies may have potentially been affected by selection bias and the use of different reference standards for infectious diseases.

## Conclusions

mNGS combined with conventional microbiological testing can improve diagnostic efficiency. We believe that mNGS may be a potential step forward in diagnosing infectious diseases due to its non-invasive, rapid and untargeted characteristics.

## Supplementary Information

Supplementary Information 1.

Supplementary Information 2.

Supplementary Information 3.

## Data Availability

The datasets used and/or analysed during the current study available from the corresponding author on reasonable request.

## Abbreviations

AUC: Area under curve
mNGS: Metagenomic next-generation sequencing
PRISMA-DTA: Preferred reporting items for a systematic review and meta-analysis of diagnostic test accuracy
NPV: Negative predictive value
PPV: Positive predictive value
CIs: Confidence intervals
sROC: A summary receiver operating characteristic curve
AUC: Area under the curve
SS: Summarise sensitivities
SP: Summarise specificities
− LR: Negative likelihood ratios
+ LR: Positive likelihood ratios
DOR: Diagnostic odds ratio
HSROC: Hierarchical summary receiver operating characteristic
PCR: Polymerase chain reaction
ID: Infectious diseases

## References

[1] Ramanan P, Bryson AL, Binnicker MJ, Pritt BS, Patel R (2018). Syndromic panel-based testing in clinical microbiology. Clin. Microbial. Rev..

[2] Barlam TF (2016). Implementing an antibiotic stewardship program: Guidelines by the infectious diseases society of America and the society for healthcare epidemiology of America. Clin. Infect. Dis..

[3] Grumaz S (2016). Next-generation sequencing diagnostics of bacteremia in septic patients. Genome Med..

[4] Goldberg B, Sichtig H, Geyer C, Ledeboer N, Weinstock GM (2015). Making the leap from research laboratory to clinic: Challenges and opportunities for next-generation sequencing in infectious disease diagnostics. mBio.

[5] Sun Z (2022). Comprehensive understanding to the public health risk of environmental microbes via a microbiome-based index. J. Genet. Genomics.

[6] Blauwkamp TA (2019). Analytical and clinical validation of a microbial cell-free DNA sequencing test for infectious disease. Nat. Microbiol..

[7] Goggin KP (2020). Evaluation of plasma microbial cell-free DNA sequencing to predict bloodstream infection in pediatric patients with relapsed or refractory cancer. JAMA Oncol..

[8] Langelier C (2018). Metagenomic sequencing detects respiratory pathogens in hematopoietic cellular transplant patients. Am. J. Respir. Crit. Care Med..

[9] Schlaberg R (2017). Viral pathogen detection by metagenomics and pan-viral group polymerase chain reaction in children with pneumonia lacking identifiable etiology. J. Infect. Dis..

[10] Shi CL (2020). Clinical metagenomic sequencing for diagnosis of pulmonary tuberculosis. J. Infect..

[11] Wilson MR (2019). Clinical metagenomic sequencing for diagnosis of meningitis and encephalitis. N. Engl. J. Med..

[12] Zhang JZ (2019). Next-generation sequencing combined with routine methods to detect the pathogens of encephalitis/meningitis from a Chinese tertiary pediatric neurology center. J. Infect..

[13] McInnes MDF (2018). Preferred reporting items for a systematic review and meta-analysis of diagnostic test accuracy studies: The PRISMA-DTA statement. JAMA.

[14] Campbell, J. *et al.* The systematic review of studies of diagnostic test accuracy. *Joanna Briggs Institute Reviewers’ Manual*, 1–46 (2015).

[15] Whiting PF (2011). QUADAS-2: A revised tool for the quality assessment of diagnostic accuracy studies. Ann. Intern. Med..

[16] Reitsma JB (2005). Bivariate analysis of sensitivity and specificity produces informative summary measures in diagnostic reviews. J. Clin. Epidemiol..

[17] Rutter CM, Gatsonis CA (2001). A hierarchical regression approach to meta-analysis of diagnostic test accuracy evaluations. Stat. Med..

[18] Higgins JP, Thompson SG (2002). Quantifying heterogeneity in a meta-analysis. Stat. Med..

[19] DerSimonian R, Laird N (1986). Meta-analysis in clinical trials. Control Clin Trials.

[20] Deeks JJ, Macaskill P, Irwig L (2005). The performance of tests of publication bias and other sample size effects in systematic reviews of diagnostic test accuracy was assessed. J. Clin. Epidemiol..

[21] Zamora J, Abraira V, Muriel A, Khan K, Coomarasamy A (2006). Meta-DiSc: A software for meta-analysis of test accuracy data. BMC Med. Res. Methodol..

[22] Moore NE (2015). Metagenomic analysis of viruses in feces from unsolved outbreaks of gastroenteritis in humans. J. Clin. Microbiol..

[23] Zhang Y (2020). Clinical application and evaluation of metagenomic next-generation sequencing in suspected adult central nervous system infection. J. Transl. Med..

[24] Miao Q (2018). Microbiological diagnostic performance of metagenomic next-generation sequencing when applied to clinical practice. Clin. Infect. Dis..

[25] Gosiewski T (2017). Comprehensive detection and identification of bacterial DNA in the blood of patients with sepsis and healthy volunteers using next-generation sequencing method - the observation of DNAemia. J. Clin. Microbiol. Infect. Dis..

[26] Rhodes J (2010). Antibiotic use in Thailand: Quantifying impact on blood culture yield and estimates of pneumococcal bacteremia incidence. Am. J. Trop. Med. Hyg..

[27] Gu W, Miller S, Chiu CY (2019). Clinical metagenomic next-generation sequencing for pathogen detection. Annu. Rev. Pathol..

[28] Xing XW (2020). metagenomic next-generation sequencing for diagnosis of infectious encephalitis and meningitis: A large, prospective case series of 213 patients. Front. Cell. Infect. Microbiol..

[29] Li H (2018). Detection of pulmonary infectious pathogens from lung biopsy tissues by metagenomic next-generation sequencing. Front. Cell. Infect. Microbiol..

[30] Wang J, Han Y, Feng J (2019). Metagenomic next-generation sequencing for mixed pulmonary infection diagnosis. BMC Pulm. Med..

[31] Boheemen SV (2019). Retrospective validation of a metagenomic sequencing protocol for combined detection of Rna and DNA viruses using respiratory samples from pediatric patients. J. Mol. Diagn. JMD.

[32] Zhang X-X (2019). The diagnostic value of metagenomic next-generation sequencing for identifying Streptococcus pneumoniae in paediatric bacterial meningitis. BMC Infect. Dis..

[33] Rossoff J (2019). Noninvasive diagnosis of infection using plasma next-generation sequencing: A single-center experience. Open Forum Infect. Dis..

[34] Miller S (2019). Laboratory validation of a clinical metagenomic sequencing assay for pathogen detection in cerebrospinal fluid. Genome Res..

[35] Madi N, Al-Nakib W, Mustafa AS, Habibi N (2018). Metagenomic analysis of viral diversity in respiratory samples from patients with respiratory tract infections in Kuwait. J. Med. Virol..

[36] Parize P (2017). Untargeted next-generation sequencing-based first-line diagnosis of infection in immunocompromised adults: A multicentre, blinded, prospective study. Clin. Microbial. Infect..

[37] Wang CX (2020). Comparison of broad-range polymerase chain reaction and metagenomic next-generation sequencing for the diagnosis of prosthetic joint infection. Int. J. Infect. Dis..

[38] Chen Y (2021). Application of metagenomic next-generation sequencing in the diagnosis of pulmonary infectious pathogens from bronchoalveolar lavage samples. Front. Cell. Infect. Microbiol..

[39] Lian QY (2021). High-throughput next-generation sequencing for identifying pathogens during early-stage post-lung transplantation. BMC Pulm. Med..

[40] Peng JM, Du B, Qin HY, Wang Q, Shi Y (2021). Metagenomic next-generation sequencing for the diagnosis of suspected pneumonia in immunocompromised patients. J. Infect..

[41] Sun T (2021). Metagenomic next-generation sequencing for pathogenic diagnosis and antibiotic management of severe community-acquired pneumonia in immunocompromised adults. Front. Cell. Infect. Microbiol..

[42] Zhou H (2021). Clinical impact of metagenomic next-generation sequencing of bronchoalveolar lavage in the diagnosis and management of pneumonia: A multicenter prospective observational study. J. Mol. Diagn..

[43] Chen H (2020). Clinical utility of in-house metagenomic next-generation sequencing for the diagnosis of lower respiratory tract infections and analysis of the host immune response. Clin. Infect. Dis..

[44] Jing C (2021). Clinical evaluation of an improved metagenomic next-generation sequencing test for the diagnosis of bloodstream infections. Clin. Chem..

[45] Ogawa S (2021). Evaluation of infections in orthopedic patients using next-generation sequencing. J. infect. Chemother..

[46] Lee RA, Al Dhaheri F, Pollock NR, Sharma TS (2020). Assessment of the clinical utility of plasma metagenomic next-generation sequencing in a pediatric hospital population. J. Clin. Microbial..

[47] Cai Y (2020). Metagenomic next generation sequencing improves diagnosis of prosthetic joint infection by detecting the presence of bacteria in periprosthetic tissues. Int. J. Infect. Dis..

