# Supplementary figures and images for: Diagnostic accuracy of metagenomic next-generation sequencing in diagnosing infectious diseases: a meta-analysis
Source: Sci Rep. 2022 Dec 5;12:21032. doi: 10.1038/s41598-022-25314-y (PMC9723114; doi:10.1038/s41598-022-25314-y)

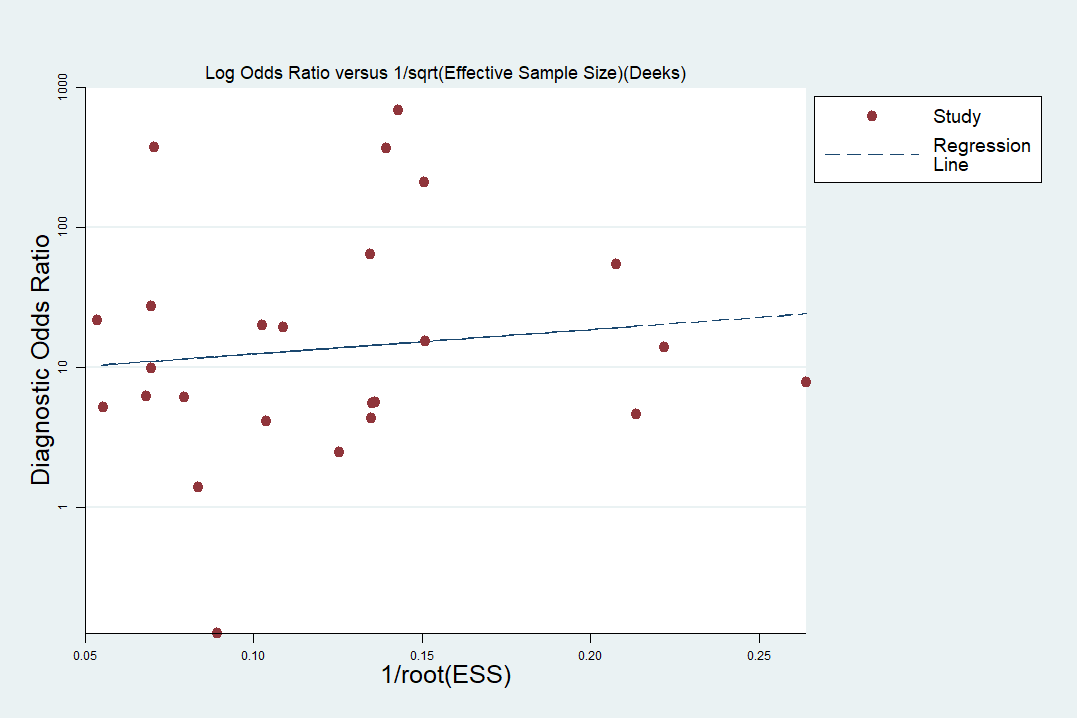

Supplement: Supplementary file 2 — Supplementary Information 2. [file 41598_2022_25314_MOESM2_ESM.tif]
