# Supplementary material for: Diagnostic accuracy of metagenomic next-generation sequencing in diagnosing infectious diseases: a meta-analysis
Source: Sci Rep. 2022 Dec 5;12:21032. doi: 10.1038/s41598-022-25314-y (PMC9723114; doi:10.1038/s41598-022-25314-y)
Supplement: Supplementary file 3 — Supplementary Information 3. [file 41598_2022_25314_MOESM3_ESM.doc]

**Diagnostic accuracy of metagenomic next-generation sequencing for diagnosis of infectious diseases: a meta-analysis**

**Jian Liu^1^*^#^,** **Qiao Zhang ^2#^,Yong-Quan Dong^3^, Jie Yin^4^, Yun-Qing Qiu^5^***

**^1^Department of Intensive Care Unit, the First Affiliated Hospital, College of Medicine, Zhejiang University, Hangzhou, Zhejiang Province, China**

**^2^Department of Clinical Pharmacy, Zhejiang Provincial Key Laboratory for Drug Evaluation and Clinical Research, the First Affiliated Hospital, College of Medicine, Zhejiang University, Hangzhou, Zhejiang Province, China**

**^3^Department of Respiratory Disease, Yinzhou No.2 Hospital, Ningbo, Zhejiang Province, China**

**^4^** **Department of Colorectal Medicine, The Cancer Hospital of the University of Chinese Academy of Sciences (Zhejiang Cancer Hospital), Institute of Basic Medicine and Cancer (IBMC), Chinese Academy of Sciences, Hangzhou, Zhejiang Province, China**

**^5^Department of Infectious Diseases, The First Affiliated Hospital, College of Medicine, Zhejiang University, Hangzhou, Zhejiang Province, China**

**^#^These authors contributed equally to this work**

***Corresponding author**

**E-mail:** [**qiuyq@zju.edu.cn**](mailto:qiuyq@zju.edu.cn)**(Yun-Qing Qiu).**

**1516050@zju.edu.cn(Jian Liu).**

**Supplementary materials:**

**Supplementary Table  Characteristics of Different Technique in NGS**

| Illumina | Its sequencing errors mainly originate from base substitution. The reading length (200bp-500bp) also limits its application. |
| --- | --- |
| Roche 454  | Cannot accurately measure PolyA. The technology introduces insertion and deletion sequencing errors in the sequencing process. |
| Ion Torrent | The throughput of microarray is not high and is very suitable for sequencing of small genomes and exon validation. |

**supplementary figure legend：**

**Supplementary Figure 1: Subgroup analysis of forest plot of estimates results: Conventional test: A. Sensitivity; B. Specificity; Clinical diagnosis: C. Sensitivity; D. Specificity.**

**Supplementary Figure 2:. Publication bias.**
